# Receptor activator of NF-κB ligand induces cell adhesion and integrin α2 expression via NF-κB in head and neck cancers

**DOI:** 10.1038/srep23545

**Published:** 2016-03-24

**Authors:** Tamaki Yamada, Masumi Tsuda, Takanori Wagatsuma, Yoichiro Fujioka, Mari Fujioka, Aya O. Satoh, Kosui Horiuchi, Shinya Nishide, Asuka Nanbo, Yasunori Totsuka, Hisashi Haga, Shinya Tanaka, Masanobu Shindoh, Yusuke Ohba

**Affiliations:** 1 Department of Cell Physiology, Hokkaido University Graduate School of Medicine, Sapporo 060-8638, Japan; 2 Division of Oral Pathobiological Science, Hokkaido University Graduate School of Dental Medicine, Sapporo 060-8586, Japan; 3 Laboratory of Oral and Maxillofacial Surgery, Hokkaido University Graduate School of Dental Medicine, Sapporo 060-8586, Japan; 4 Department of Cancer Pathology, Hokkaido University Graduate School of Medicine, Sapporo 060-8638, Japan; 5 Transdisciplinary Life Science Course, Faculty of Advanced Life Science, Hokkaido University, Sapporo 060-0810, Japan

## Abstract

Cellular interactions with the extracellular matrix play critical roles in tumor progression. We previously reported that receptor activator of NF-κB ligand (RANKL) specifically facilitates head and neck squamous cell carcinoma (HNSCC) progression *in vivo*. Here, we report a novel role for RANKL in the regulation of cell adhesion. Among the major type I collagen receptors, integrin α2 was significantly upregulated in RANKL-expressing cells, and its knockdown suppressed cell adhesion. The mRNA abundance of integrin α2 positively correlated with that of RANKL in human HNSCC tissues. We also revealed that RANK-NF-κB signaling mediated integrin α2 expression in an autocrine/paracrine manner. Interestingly, the amount of active integrin β1 on the cell surface was increased in RANKL-expressing cells through the upregulation of integrin α2 and endocytosis. Moreover, the RANK-integrin α2 pathway contributed to RANKL-dependent enhanced survival in a collagen gel and inhibited apoptosis in a xenograft model, demonstrating an important role for RANKL-mediated cell adhesion in three-dimensional environments.

Head and neck squamous cell carcinoma (HNSCC) constitutes the sixth most common malignancy worldwide^1^. Despite therapeutic and diagnostic advances, the 5-year survival rate for this disease remains at approximately 50%, due to a high degree of local invasiveness and metastasis to cervical lymph nodes^2^. It has been widely accepted that such malignant characteristics are a consequence of communication between cancer cells and their cognate environment. Moreover, given that the head and neck region is an organ that is challenged by a wide variety of environmental irritants, it is particularly important to consider the effect of the microenvironment on the development and promotion of HNSCC for a better understanding of the pathogenesis and for the establishment of efficient therapeutics. Indeed, due to a lack of such therapeutic strategies, HNSCC treatment largely depends on radical surgery, which results in significant functional and cosmetic defects as well as a significant reduction in patient quality of life. Therefore, to improve patient quality of life, it is essential to develop sensitive and reliable biomarkers that predict aggressive HNSCC and eventually provide the most suitable (i.e., necessary and sufficient) treatment for each individual patient^3^. Unfortunately, there are currently few available biomarkers that satisfy these criteria^4^.

The extracellular matrix (ECM) is one of the first environmental factors encountered by epithelial cancer cells migrating from the tumor mass. These cancer cells interact with the surrounding ECM through integrins, a family of primary ECM receptors. Integrins are required for a wide variety of cellular processes, including cell growth and differentiation, tissue repair, intracellular signaling, and tumorigenesis^5^,^6^,^7^. To date, EGFR activation has been demonstrated to be critical for integrin α2-mediated adhesion and motility in colon cancer^8^. Indeed, several studies have demonstrated the significance of adhesion between cancer cells and the ECM^9^,^10^. The expression and distribution patterns of integrin family members have also been investigated in oral, pancreatic, breast, lung, and colon cancers^7^,^11^,^12^,^13^,^14^, and integrins have been shown to play significant roles in malignant tumor invasion, migration, and metastasis^15^,^16^,^17^.

We recently reported that receptor activator of NF-κB ligand (RANKL) expression induces epithelial mesenchymal transition (EMT) and angiogenesis in HNSCC *in vivo* and correlates with histological differentiation in human HNSCC specimens^18^. Despite such aggressive phenotypes *in vivo*, RANKL could not enhance either cell proliferation or cell migration/invasion, indicating that RANKL is a specific marker of tumor progression *in vivo*. In this study, we investigated the detailed molecular mechanisms by which RANKL evokes malignant phenotypes and unveiled a previously unknown function of RANKL in the regulation of cell adhesion. RANKL elicited RANK-NF-κB signaling to upregulate integrin α2 expression and subsequent cell adhesion to type I collagen by facilitating active integrin trafficking. The enhanced cell adhesion resulted in increased survival in collagen-rich environments, including a collagen gel and tumor tissues with a poorly differentiated phenotype *in vivo*. Furthermore, RANKL and integrin α2 expression levels were significantly correlated in human oral cancer tissues. These findings highlight that RANKL and its downstream signaling may be functional biomarkers and, presumably, attractive therapeutic targets in HNSCC.

## Results

### Enhanced adhesion of RANKL-expressing cells to type I collagen via integrin α2

**Figure 1:**
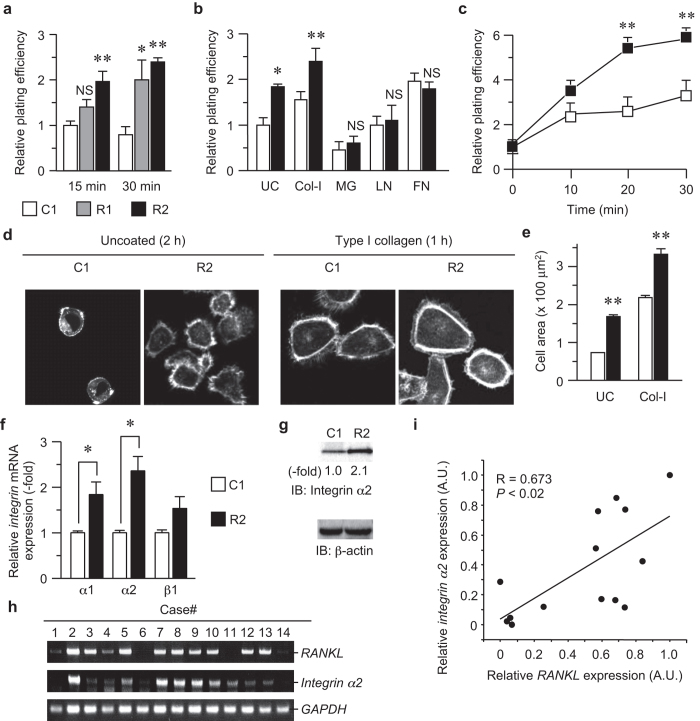
RANKL enhanced cell adhesion to type I collagen via integrin α2. (**a**) C1, R1, and R2 cells were plated on type I collagen-coated 96-well dishes at a density of 4 × 10^5^ cells/well. After 15 or 30 min, the medium was removed, and the adherent cells were stained with 0.04% crystal violet, followed by quantification by measuring the absorbance at 595 nm. *P < 0.01; **P < 0.005; NS, not significant. (**b**) C1 and R2 cells were plated on dishes coated with the indicated matrices or left uncoated (UC). After 30 min, the adherent cells were quantified as in (**a**). Col-I, type I collagen; MG, Matrigel; LN, laminin; FN, fibronectin; *P < 0.01; **P < 0.005; NS, not significant. (**c**) Cell adhesion to type I collagen was determined at the indicated time points as described in (**a**,**b**). **P < 0.005. (**d**,**e**) C1 and R2 cells were plated on uncoated and collagen-coated slides for 2 h and 1 h, respectively. F-actin was visualized by AlexaFluor 594-conjugated phalloidin. The cells were observed with a laser scanning confocal microscope (**d**). The cell area in each image was calculated using image-processing software and plotted. **P < 0.005 (**e**). (**f**) The mRNA expression levels of *integrin α1*, *α2*, and *β1*were determined by real-time quantitative PCR. The data were normalized to the *GAPDH* expression level, and the expression levels relative to those in C1 cells are shown. *P < 0.01. (**g**) The integrin α2 protein levels in C1 and R2 cells were analyzed by immunoblotting with an anti-integrin α2 antibody. (**h**,**i**) The total RNA was isolated from the tumors from the HNSCC patients and subjected to conventional RT-PCR analysis (**h**). The expression levels of the *RANKL* and *integrin α2* mRNAs were plotted, and the correlation between them was evaluated by Pearson’s product-moment correlation coefficient (**i**).

We previously reported that RANKL expression enhances tumor formation in mice^18^. To gain insight into the mechanism of the enhanced tumor formation, we established HNSCC cell line HSC-derived cells that stably express RANKL, and evaluate a variety of cellular functions involved in tumor formation/promotion. Nevertheless, although there was a dramatic increase in tumor formation by RANKL-expressing cells *in vivo*, we have not observed a difference in the *in vitro* phenotypes, including proliferation, motility, and invasion, between RANKL-expressing cells (R1 and R2 cells, hereafter) and control cells (C1 cells)^18^. We therefore postulated that the accelerated tumor growth by RANKL-expressing cells could be due to altered cell-matrix interactions because the integrin family of heterodimeric receptors for the extracellular matrix is involved in a range of processes related to tumor promotion^5^,^19^,^20^. Indeed, RANKL expression enhanced cellular adhesion to type I collagen and uncoated dishes over time but did not promote adhesion to the other tested matrices (Fig. 1a–c). In agreement with this potentiated adhesion, cell spreading after attachment to either the uncoated or collagen-coated dishes was facilitated by RANKL expression (Fig. 1d,e), indicating that cell adhesion-induced signaling is also activated in RANKL-expressing cells.

Next, to explore the mechanism by which RANKL enhanced cell adhesion, we examined the expression levels of the cell surface collagen receptors, namely integrin α1, α2, and β1, the combinations of which (α1/β1 and α2/β1) are known to dictate cell-to-collagen interactions^21^. As shown in Fig. 1f, all of these integrins were expressed more abundantly in the RANKL-expressing cells than in the control cells, and integrin α2 level showed the most significant increase among them. Therefore, we focused on integrin α2 for all of the subsequent experiments. In fact, integrin α2 protein expression was also increased by approximately two-fold in the RANKL-expressing cells (Fig. 1g). Moreover, *integrin α2* mRNA expression positively correlated with *RANKL* expression in surgically resected human HNSCC specimens (Fig. 1h,i).

**Figure 2:**
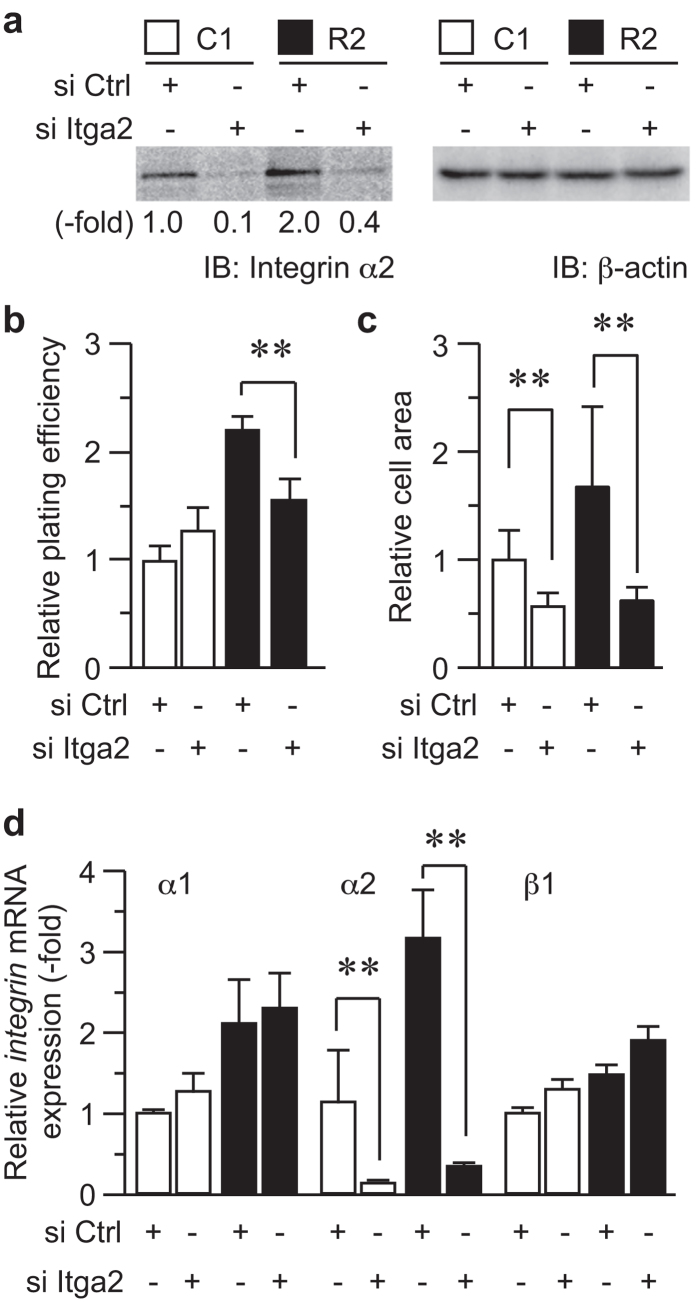
Integrin α2 mediates RANKL-dependent cell adhesion. (**a**) C1 and R2 cells were transfected with an siRNA against integrin α2 (si Itga2) or a scrambled siRNA (si Ctrl) as a negative control. After 72 h, the levels of the integrin α2 protein were determined by immunoblotting. The levels of β-actin are shown as a loading control. (**b**) C1 and R2 cells were prepared as in (**a**) and plated on collagen-coated dishes, and cell adhesion was determined as described as in Fig. 1a. **P < 0.005. (**c**) C1 and R2 cells were prepared as in (**a**) and plated on collagen-coated dishes, and the cell area was determined as in Fig. 1d. **P < 0.005. (**d**) C1 and R2 cells were prepared as in Fig. 1a and subjected to real-time quantitative PCR analysis to determine the expression levels of the *integrin α1*, *α2*, and *β1* mRNAs. **P < 0.005.

To determine whether the integrin α2 upregulation was causatively involved in RANKL-dependent cell adhesion, its expression was knocked down by a small interfering RNA (siRNA) against integrin α2 (si Itga2). Transfection of si Itga2 successfully reduced integrin α2 protein expression by approximately 90% (Fig. 2a). Under this experimental condition, the knockdown of endogenous integrin α2 partially and completely repressed the RANKL-enhanced adhesion to type I collagen-coated dishes (Fig. 2b) and cell spreading on the dishes (Fig. 2c), respectively. Given that knockdown of integrin α2 resulted in only partial inhibition of adhesion to type I collagen, RANKL may also promote cell adhesion via an unknown mechanism, which may account for enhancement of cell adhesion on uncoated dishes. On the other hand, the knockdown did not affect the levels of integrin α1 and β1 (Fig. 2d), integrin α2 dictated RANKL-dependent cell adhesion among integrins serving as the collagen I receptor.

### Requirement for NF-κB in RANKL-dependent upregulation of integrin α2 expression and cell adhesion

**Figure 3:**
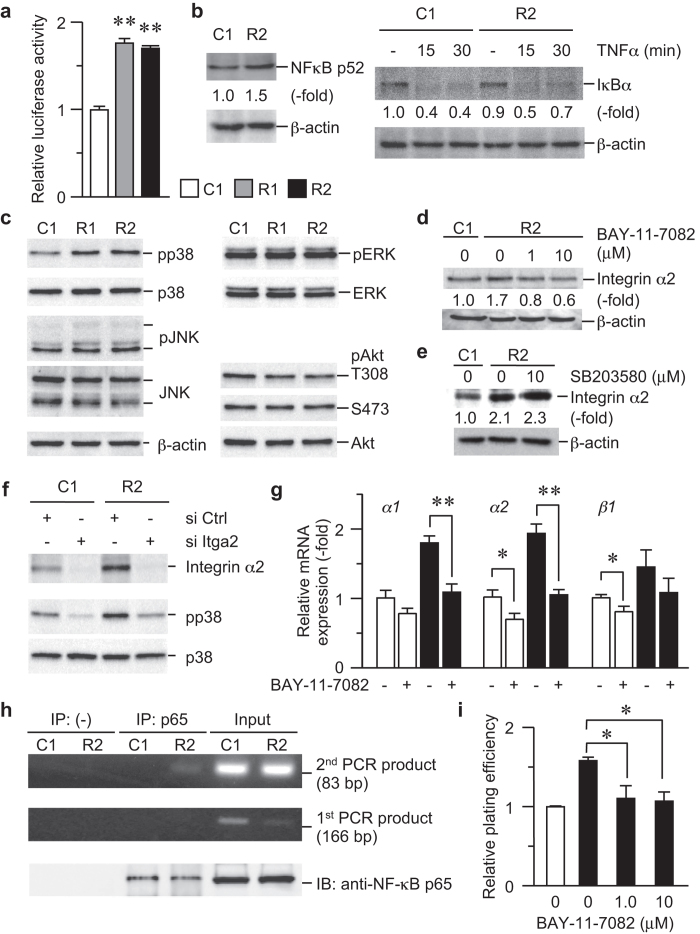
RANKL-dependent integrin α2 expression via NF-κB. (**a**) C1, R1, and R2 cells were transfected with pRT-TK-Luc and pNF-κB-Luc. The luciferase activities were measured after 24 h. The *Firefly* luciferase activity in each sample was normalized to that of the *Renilla* luciferase activity. The luciferase activity in the RANKL-expressing cells was further normalized to that in C1 cells, and the relative activity was plotted. **P < 0.005. (**b**) C1 and R2 cells were treated with TNF-α or left untreated. The levels of p52 (left panels) and IκBα (right panels) were analyzed by immunoblotting. β-actin was used for a loading control. (**c**) The lysates from the indicated cells were subjected to immunoblotting analysis to determine phosphorylation levels of downstream signaling mediators of RANKL, namely p38, JNK, ERK, and Akt. The total protein and β-actin levels are also shown. (**d**) R2 cells were treated with DMSO or 1.0 or 10.0 μM BAY-11-7082, an NF-κB inhibitor, for 72 h. The levels of integrin α2 were analyzed by immunoblotting. C1 cells were used as a control. (**e**) C1 and R2 cells were treated with SB203580 or left untreated for 16 h. The levels of integrin α2 were analyzed by immunoblotting. C1 cells were used as a control. (**f**) C1 and R2 cells were transfected with si Ctrl or si Itga2, and, after 72 h, the p38 phosphorylation and total protein levels, as well as the levels of the integrin α2 protein, were determined by immunoblotting. (**g**) C1 and R2 cells were treated with BAY-11-7082 or left untreated. The levels of indicated integrin were determined by real-time quantitative PCR and are expressed as the fold change compared with untreated C1 cells. *P < 0.01; **P < 0.005. (**h**) C1 and R2 cells were fixed with 1% formaldehyde and lysed. The nuclear fraction was sonicated to shear chromatin and immunoprecipitated with an anti-NF-κB p65 antibody. p65-coprecipitating DNA was analyzed by nested PCR with promoter-specific primers amplifying the *Itga2* promoter. (**i**) C1 and R2 cells were treated with the indicated concentrations of BAY-11-7082 or left untreated for 48 h. The cells were trypsinized and then plated on type I collagen-coated dishes. After 30 min, the adherent cells were quantified as in Fig. 1a. *P < 0.01.

We further examined the activity of the possible downstream factors of RANKL and found that NF-κB was activated in the RANKL expressing cells (Fig. 3a). The non-canonical NF-κB pathway, but not the canonical pathway, might be activated in RANKL-expressing cells because the amount of NF-κB p52 were upregulated in RANKL-expressing cells, whereas the level of IκBα was not altered between control and R2 cells (Fig. 3b). We also analyzed the activity of mitogen-activated protein kinase pathways and other pathways and found that p38 mitogen-activated protein kinase (p38; Fig. 3c) were selectively activated in the RANKL-expressing cells, whereas other candidates, including c-Jun N-terminus kinase (JNK), extracellular signal-regulated kinase (ERK) and Akt, were not activated (Fig. 3c). We further examined the expression level of integrin α2 upon pharmacological inhibition of NF-κB and p38. Treatment with the NF-κB inhibitor BAY-11-7082 (Fig. 3d), but not the p38 inhibitor SB203580 (Fig. 3e), repressed integrin α2 expression (by ~65%), which is consistent with previous reports suggesting that NF-κB regulates cell adhesion by activating integrin α2 transcription^22^,^23^,^24^,^25^. On the other hand, integrin α2 knockdown resulted in a significant decrease in p38 phosphorylation, but not expression, indicating that p38 activity is regulated downstream of integrins (Fig. 3f). Again, this result agrees with the previous reports in which p38 is activated by integrins and focal adhesion kinase in endothelial cells exposed to shear stress^26^,^27^. BAY11-7082 treatment inactivated *integrin α2* transcription (in addition to that of integrin α1; Fig. 3g), indicating that NF-κB regulates integrin α2 expression at the transcription level. In fact, a chromatin immunoprecipitation assay demonstrated that more NF-κB bound to the promoter region of the *Itga2* gene (Fig. 3h). BAY11-7082 treatment also diminished cell adhesion to type I collagen-coated dishes (by approximately 30%; Fig. 3i). These results together implicate NF-κB as a transcriptional mediator of RANKL-dependent cell adhesion.

### RANKL functions via its receptor RANK

**Figure 4:**
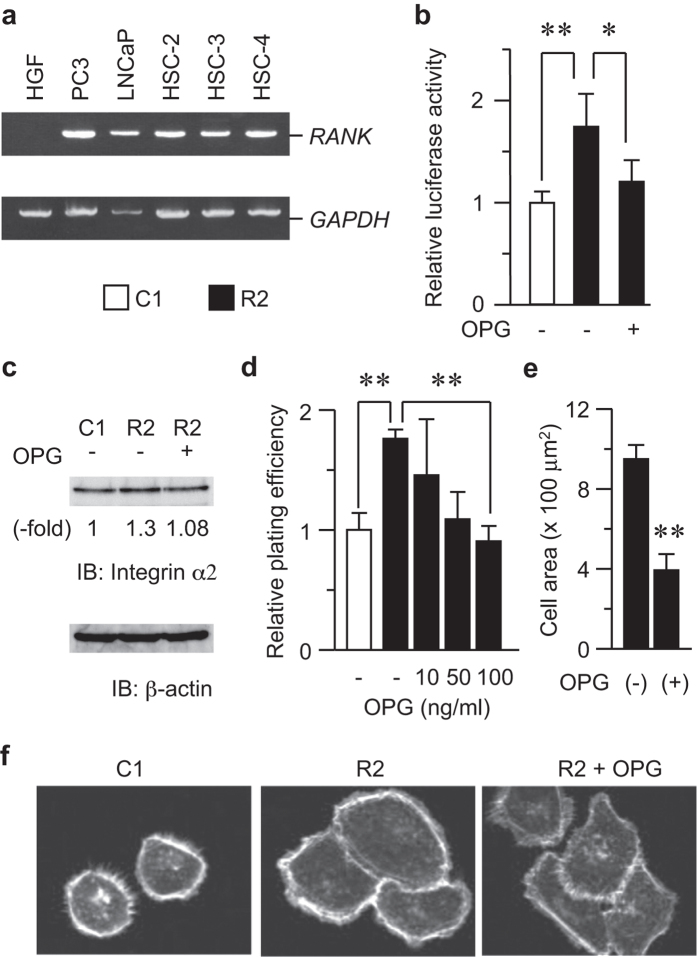
RANKL induced cell adhesion via its receptor RANK. (**a**) The levels of the *RANK* mRNA in the cells indicated were determined by RT-PCR. Note that the PC3 and LNCaP prostate cancer cell lines were used as positive controls, whereas human gingival fibroblasts (HGF) were used as a negative control. (**b**) R2 cells were treated with 100 ng/ml of the RANKL decoy receptor osteoprotegerin (OPG) or left untreated for 48 h. The promoter activity of NF-κB was determined as in Fig. 3a. C1 cells were used as a control. *P < 0.01; **P < 0.005. (**c**) R2 cells were treated with or without 100 ng/ml of OPG for 72 h. The integrin α2 protein levels were analyzed by immunoblotting. C1 cells were used as a control. (**d**) R2 cells were treated with the indicated concentrations of OPG or left untreated. After 72 h, cell adhesion was determined as described above. C1 cells were used as a control. **P < 0.005. (**e**,**f**) R2 cells were treated with 100 ng/ml of OPG for 72 h, fixed, and stained with AlexaFluor 594-conjugated phalloidin. The cell area was determined by confocal microscopy and image-processing software. **P < 0.005 (**e**). Representative images are shown. C1 cells were used as a control (**f**).

During the course of our experiments, we noticed that HNSCC cell lines expressed the RANKL receptor RANK (Fig. 4a) as abundantly as the PC3 and LNCaP cell lines from prostate cancer bone metastases, which are known to express this molecule^28^,^29^. This result suggested that RANKL may act in an autocrine or paracrine manner in HNSCC. To confirm the autocrine/paracrine function of RANKL in HNSCC, we utilized the soluble RANKL decoy receptor osteoprotegerin (OPG), which inhibits the RANKL-RANK pathway by sequestering RANKL^30^,^31^,^32^,^33^. OPG inhibited NF-κB activity (after 24 h, Fig. 4b) and integrin α2 expression (after 72 h, Fig. 4c) in RANKL-expressing HNSCC cells by approximately 30%, which was comparable to the control cells. In addition, HNSCC cell adhesion to type I collagen was also suppressed by OPG in a dose-dependent manner and was comparable to that of the control cells at 100 ng/ml (Fig. 4d). Moreover, OPG also restored cell size to that of control cells (Fig. 4e,f).

### The RANKL-NF-κB-integrin α2 pathway upregulates active integrins at the cell surface

**Figure 5:**
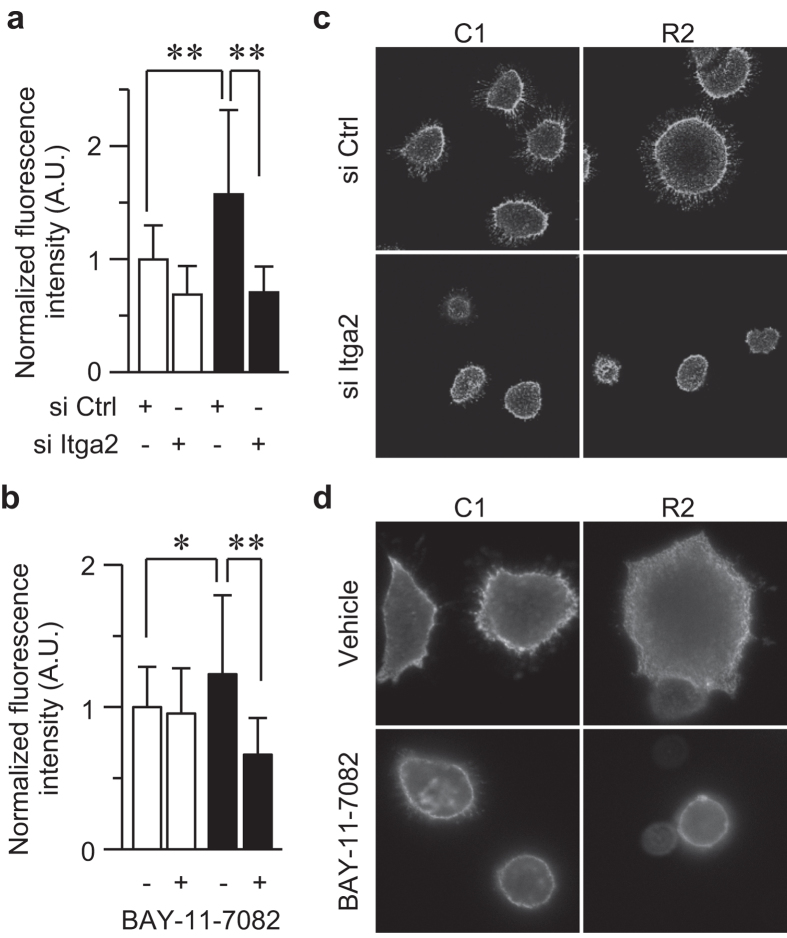
The RANKL-integrin α2 axis upregulates the amount of active integrin on the cell surface. (**a**,**c**) C1 and R2 cells were transfected with indicated siRNA oligonucleotides. After 72 h, the cells were replated on collagen-coated dishes for 1 h and directly incubated with an anti-active integrin β1 antibody (AIIB2) for 45 min on ice, followed by visualization with an AlexaFluor 488-conjugated secondary antibody. After fixation, the cells were observed under a fluorescence microscope, and the fluorescence intensities were plotted as the means with S.D. **P < 0.005 (**a**). Representative images are shown (**c**). (**b**,**d**) C1 and R2 cells were treated with 1.0 μM of the NF-κB inhibitor BAY-11-7082 or left untreated for 72 h, and subjected to live cell immunostaining as in (**a**). *P < 0.01; **P < 0.005 (**b**). Representative images are shown (**d**).

To gain further insight into how RANKL-evoked signaling regulates cell adhesion, integrin activation was analyzed using the active integrin β1 specific-antibody (AIIB2). When living, non-permeabilized cells were stained with this antibody, the expression of active integrin β1 was increased at the surface of the RANKL-expressing cells (Fig. 5a,b). The upregulation was restored by si Itga2 (Fig. 5a), which and was accompanied by decreased cell size (Fig. 5c). Because the siRNA targeting integrin α2 specifically repressed the expression of integrin α2 without affecting integrin β1 (Fig. 2d), the decreased level of active integrin β1 was due to the decrease in integrin α2, but not integrin β1 itself. Similarly, BAY-11-7082 also inhibited the active integrins at the cell surface (Fig. 5b,d), confirming that NF-κB plays a role in RANKL-mediated upregulation of active integrins on the cell surface.

### RANKL upregulates endocytosis

**Figure 6:**
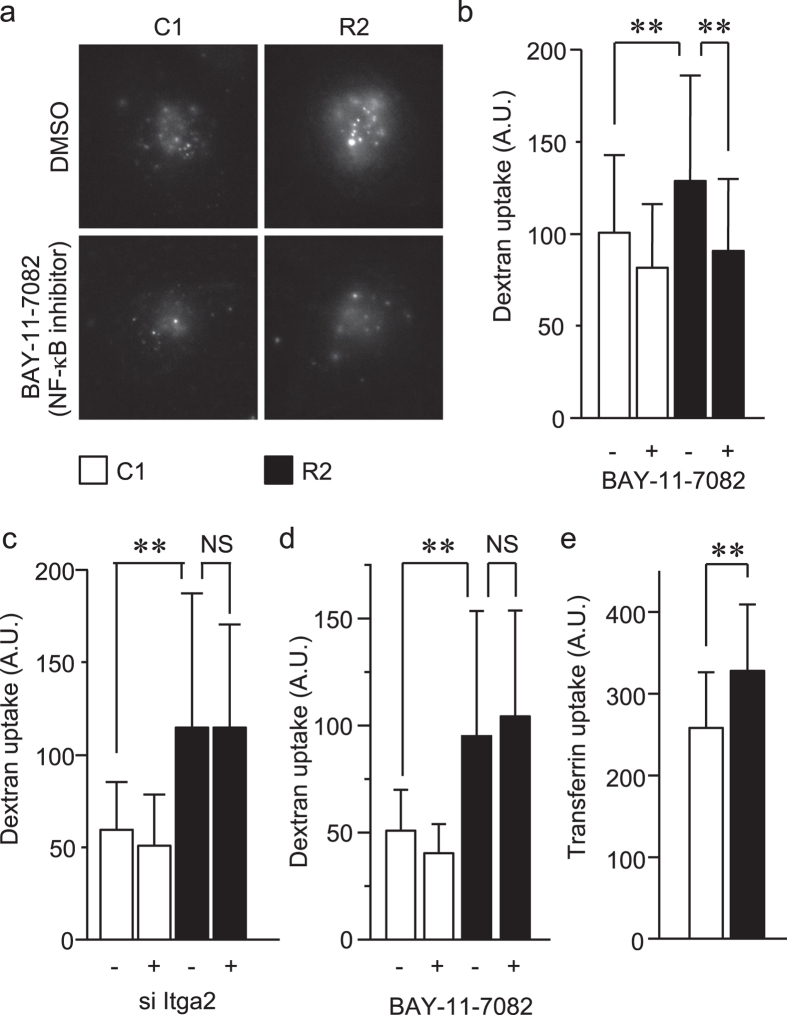
RANKL enhanced endocytosis in a NF-κB-dependent and integrin α2-independent manner. (**a**) C1 and R2 cells were treated with 5 μM of the NF-κB inhibitor BAY-11-7082 or vehicle alone (DMSO) for 72 h and then incubated with 0.5 mg/ml of Alexa Fluor 546-conjugated dextran for 10 min. After washing with PBS, the cells were observed under a fluorescence microscope. Representative images are shown. (**b**) Using the images obtained in (**a**), the fluorescence intensity of the cells were quantified and plotted as the amount of incorporated dextran. **P < 0.005. (**c**) C1 and R2 cells were transfected with siRNA against integrin α2 for 72 h and subjected to the dextran uptake assay as in (**a**,**b**). **P < 0.005; NS, not significant. (**d**) C1 and R2 cells were treated with 5 μM of the NF-κB inhibitor BAY-11-7082 or vehicle alone (DMSO) for 30 min and then subjected to the analysis as in (**a**,**b**). **P < 0.005; NS, not significant. (**e**) Activities of clathrin-dependent endocytosis in C1 and R2 cells were evaluated using Alexa Fluor 546-conjugated transferrin. **P < 0.005.

It has been reported that endocytic trafficking of the active integrin complex plays critical roles in regulating the cell-to-ECM interaction^34^,^35^,^36^, which prompted us to evaluate whether RANKL facilitated endocytosis. Fluorescently labeled dextran was utilized to evaluate fluid-phase, clathrin-independent endocytosis by measuring the fluorescence intensities corresponding to substrate uptake. Dextran uptake (i.e., clathrin-independent endocytosis) was significantly increased by approximately two-fold in the RANKL-expressing cells (Fig. 6a,b). This upregulation was restored to basal levels by the NF-κB inhibitor (Fig. 6a,b) but not by integrin α2 knockdown (Fig. 6c). Interestingly, treatment with BAY-11-7082 for shorter periods (30 min) failed to inhibit the RANKL-dependent upregulation of endocytosis (Fig. 6d), which, together, indicate that transcriptional upregulation of NF-κB target molecule(s) other than integrin α2 dictate the RANKL-dependent upregulation of endocytosis. Because transferrin uptake was also upregulated in the RANKL-expressing cells, RANKL was also able to facilitate clathrin-dependent endocytosis to some extent (Fig. 6e).

### The RANKL-NF-κB-integrin α2 pathway regulates integrin trafficking

**Figure 7:**
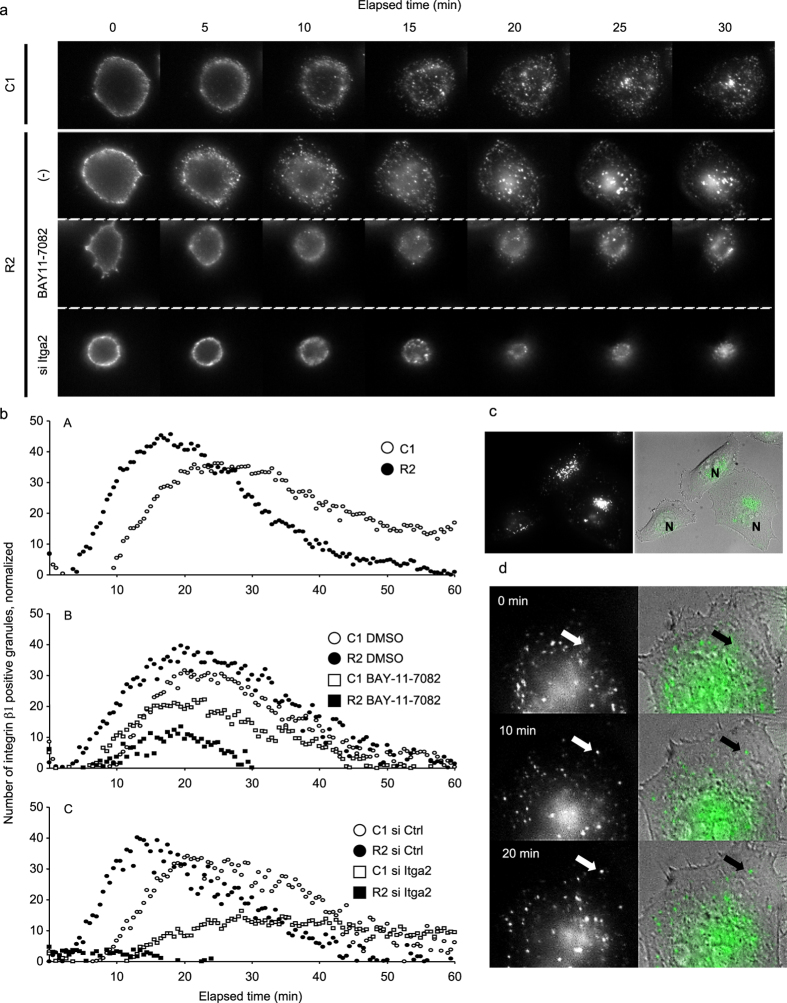
The RANKL-integrin α2 axis regulates integrin trafficking. (**a**) The following cells were trypsinized, incubated with an anti-active integrin β1 antibody, and further incubated with an AlexaFluor 488-conjugated secondary antibody: C1 cells, R2 cells, R2 cells treated with the NF-κB inhibitor BAY11-7082 for 24 h, and R2 cells transfected with si Itga2 for 72 h. The cells were then re-plated on collagen-coated glass-bottom dishes and subjected to time-lapse fluorescence microscopy. Representative images are shown (see also Suppl. Mov. 1, S). (**b**) The number of intracellular granules containing active integrin β1 at each time point was calculated using image-processing software and plotted for C1 and R2 cells (A), C1 and R2 cells treated with the NF-κB inhibitor or DMSO (B), and C1 and R2 cells transfected with si Itga2 (C). (**c**) Representative fluorescence (left) and merged (right) images of C1 cells that were treated as in (**a**). N, nucleus. (**d**) Enlarged view of serial images over a 20-min period, with 10-min intervals. The left and right panels are the fluorescence and merged fluorescence and phase-contrast images. The arrows indicate the integrin-positive granules that moved from the central region to the peripheral region of the cells.

As described above, RANKL-NF-κB signaling upregulates the amount of integrin β1 on the cell surface without affecting its total amount (Fig. 1f). Thus, we observed active integrin transport in living cells. The trypsinized cells were treated with trypsin inhibitor, chilled on ice, and directly incubated with the AIIB2 antibody, followed by visualization with an AlexaFluor 488-conjugated secondary antibody. As shown in Fig. 7a,bA, active integrin β1 was internalized as early as 10 min after replating. The number of granules visualized by the antibody reached a maximum value at approximately 25–30 min and then decreased gradually in the control cells (See also Suppl. Mov. 1). At the late stage of the observation period, the fluorescence signals were accumulated in the specific perinuclear region of the cells, which may be the perinuclear recycling endosomes (Fig. 7c). RANKL expression clearly enhanced the timing and extent of integrin endocytosis. The increased internalization began at 5 min, the zenith emerged at 15 min, and the number of vesicles was increased by approximately 1.5-fold (Fig. 7a,bA; Suppl. Mov. 2). Both the NF-κB inhibitor (Fig. 7a,bB) and integrin α2 knockdown (Fig. 7a,bC) blocked the internalization of active integrin β1, indicating that these molecules contribute to cell adhesion by regulating integrin trafficking. In addition, during the observation period, a significant number of granules moved to the peripheral region of the cells (Fig. 7d, arrow), which might reflect the process of integrin recycling to the plasma membrane.

### RANKL promotes increased viability in an ECM-enriched environment

**Figure 8:**
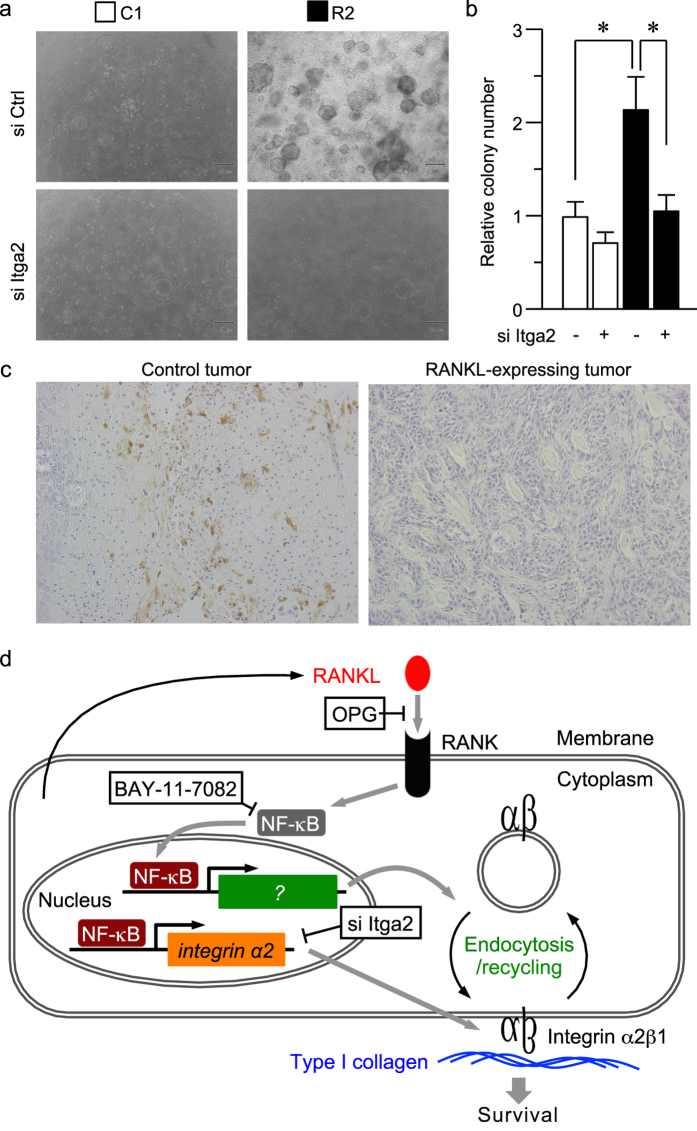
The RANKL-integrin α2 axis enhances cell survival in the collagen-rich environment. (**a**,**b**) C1 and R2 cells were transfected with an siRNA nucleotide against integrin α2 (si Itga2) or the control siRNA (si Ctrl). After 48 h, the cells were seeded on 12-well plates at a density of 2 × 10^4^ cells per well in 0.3 mg/ml collagen containing DMEM and cultured for 21 days. Representative images are shown (**a**). The number of colonies formed were counted and plotted. *P < 0.01 (**b**). (**c**) The tumor tissue sections of xenograft tumors derived from C1 and R2 cells were subjected to deoxynucleotidyl transferase-mediated nick-end labeling (TUNEL) staining. (**d**) Schematic summarization of the current study. RANKL functions in an autocrine manner through its cognate receptor RANK. RANKL-RANK binding evokes intracellular signaling, including NF-κB signaling. Activated NF-κB, in turn, induces the transcription of integrin α2 and gene(s) involved in the regulation of endocytosis. This signaling pathway upregulates the amount of active integrin α2β1 on the cell surface and promotes cell adhesion to type I collagen, which enhances cell survival in 3D matrices.

We previously reported that RANKL-expressing cells display a more invasive morphology [epithelial branching, an EMT-dependent event^37^] in collagen gel than control cells^18^. Therefore, similar sets of experiments were performed to evaluate the significance of RANKL-NF-κB-integrin α2 signaling in a collagen-enriched environment. RANKL-expressing cells exhibited a more efficient colony formation than control cells (Fig. 8a,b). The increased colony formation was repressed by si Itga2, indicating that the RANKL-enhanced colony formation in a type I collagen gel was mediated by integrin α2 (Fig. 8a,b). The increase in colony formation may be partially due to suppressed apoptosis, which may contribute to the RANKL-enhanced tumorigenesis^18^. In fact, there were no deoxynucleotidyl transferase-mediated nick-end labeling (TUNEL) staining-positive cancer cells observed in the tumors formed by RANKL-expressing cells in a mouse xenograft model, whereas such cells were abundant in the control tumors (Fig. 8c).

## Discussion

In the present study, we identified a novel RANKL function in the promotion of cell adhesion to type I collagen through the RANKL-RANK-NF-κB-integrin α2 pathway (Fig. 8d). The activation of this signaling pathway resulted in enhanced cell adhesion and survival in a collagen-rich environment through the upregulation of integrin trafficking and the amount of active integrins on the cell surface. Therefore, the increased integrin α2 expression and cell adhesion may play a role, at least in part, in the RANKL-induced tumorigenesis *in vivo*^18^. We also demonstrated that RANKL mediates cell adhesion to type I collagen through NF-κB and integrin α2. Thus, our data provide a bridge between the described NF-κB functions of tumor promotion^38^,^39^ and consolidated cell adhesion^22^,^23^,^24^,^25^.

Tumorigenesis is a biological cascade of multiple steps, including cell adhesion, invasion, migration, and uncontrolled cell growth. Among these crucial steps in epithelial cancer cells, adhesion to ECM proteins, which is mediated by members of the integrin family, is not only an important determinant of organized growth and the maintenance of architectural integrity in a tumor mass, but is also the first step for invasion into the surrounding tissue by the cells emigrating from the mass. Thus, changes in cell-ECM adhesion accompany the conversion from benign tumors to invasive, malignant cancers and the subsequent metastatic dissemination of tumor cells^40^. RANKL-expressing cells promoted cellular adhesion to collagen (Fig. 1), but failed to promote cell invasion, migration, and cell growth *in vitro*^18^. These results strongly implicate RANKL-induced cell adhesion in tumorigenesis *in vivo*.

The cognate RANKL receptor RANK is expressed as abundantly in HNSCC as in cell lines from prostate cancer bone metastases (Fig. 4). Given that OPG treatment inhibited the RANKL-dependent upregulation of NF-κB transcriptional activity, integrin α2 expression, and subsequent cell adhesion, RANKL may function, at least in part, in a paracrine/autocrine manner via RANK in HNSCC. In addition, the mechanism of action of RANKL via its receptor RANK may be conserved between bone and head and neck cancer. Similar variations in osteoclast signaling have been observed in several bone metastases, e.g., from breast and prostate cancers^28^,^29^,^41^,^42^,^43^,^44^, in which RANKL expression is completely depends on the bone stroma. Various cytokines, such as parathyroid hormone (PTH), promote RANKL expression through the canonical regulatory mechanism in osteoblasts^45^. Similarly, EGF and the subsequent PTHrP production^46^ partially contribute to the augmented RANKL expression in HNSCC in the head and neck environment. Currently, stimulations with these factors have failed to induce RANKL expression *in vitro*.

It has been well established that RANKL binds to its receptor RANK to transduce signals into cells by recruiting various adaptor proteins, including TNFR-associated factors (TRAFs), and activating JNK, ERK, p38, NFATc1, Akt, and NF-κB^47^,^48^,^49^,^50^,^51^. While we did not observe striking phosphorylation of JNK, ERK, or Akt, the proteins NF-κB and p38 were activated in RANKL-expressing cells. The analysis of the 5′-flanking region of the α2 integrin gene (*Itga2*) revealed that several transcription factor binding sites, including AP-1, Sp1, and NF-κB sites, are present in the promoter region^52^. It has been indeed shown that NF-κB regulates integrin α2 expression and cell adhesion^53^. The NF-κB inhibitor BAY-11-7082 consistently and completely abolished RANKL-dependent integrin α2 upregulation (Fig. 3), confirming that NF-κB is the major effector that transduces the RANKL-mediated cell adhesion signaling. On the other hand, p38 is also activated by RANKL in HNSCCs, but its activation was downstream of integrin signaling (Fig. 3).

Several lines of evidence have suggested that the integrin expression profile may serve as a tool to predict the prognosis of individual patients. For instance, the expression levels of integrins α5, β1, and β3 predicted the overall and disease-free survival of early stage non-small cell lung cancer patients^54^. In addition, the expression levels of integrins α2, β4, and β5, with functional normalization by desmosomal or cytoskeletal molecule genes, were selected as candidate biomarkers for cervical LN metastasis, which is related to mortality in HNSCC patients^55^. On the other hand, RANKL also serves as a potential risk factor in prostate cancer^56^ and head and neck cancer patients^18^. Given that the integrin α2 expression positively correlated with RANKL expression in HNSCC patient samples (Fig. 1), RANKL and its downstream signaling molecule integrin α2 may be functional markers for aggressive HNSCC.

The significance of RANKL becomes more apparent in conditions that more closely approximate the *in vivo* situation. The RANKL-expressing cells increased cell growth in a three-dimensional (3D) gel (Fig. 8), and the RANKL-expressing tumors did not exhibit TUNEL staining compared with the control tumors (Fig. 8). These RANKL functions may be attributed to the upregulation of integrin expression. Cell adhesion via integrins is well known to protect cells against apoptosis^57^ and malignant transformation^54^,^56^. Recently, integrins have been shown to play a role in cancer cell escape from anoikis^58^,^59^,^60^,^61^,^62^,^63^. These experimental data provide convincing evidence that RANKL and its downstream molecule integrin α2 promote the escape from anoikis. It has also been reported that the Core3 *O*-glycan synthase suppresses prostate tumor formation and metastasis by downregulating the α2β1 integrin complex^57^. Notably, the PC3 and LNCaP cell lines used in that study also express RANK (Fig. 4); we can thus assume that the RANK-mediated regulation of integrin α2 is diminished by Core3 synthase expression, which leads to repressed tumor formation. Therefore, it appears likely that RANKL-RANK signaling to integrins is conserved among cancer types and is essential for tumorigenesis and malignant conversion in a type I collagen-enriched microenvironment via escape from anoikis. In summary, our results suggest the fascinating possibility that RANKL and its downstream signaling molecule, integrin α2, may be indicators of tumorigenesis in HNSCC and are attractive functional markers for this malignancy.

## Methods

### Cell culture

The HSC-2 (JCRB0622), HSC-3 (JCRB0623), and HSC-4 (JCRB0624) human head and neck squamous cell carcinoma (HNSCC) cell lines were obtained from the Japanese Collection of Research Bioresources (JCRB) cell bank (Osaka, Japan). Human gingival fibroblasts (HGF, CRL-1740) and the PC3 (CRL-1435) and LNCaP (CRL-1740) human prostate cell lines were purchased from the American Type Culture Collection (ATCC, Manassas, VA, USA). The cells used in this study were maintained in Dulbecco’s modified Eagle’s medium (Sigma, St. Louis, MO, USA) supplemented with 10% fetal bovine serum (FBS; Invitrogen, Carlsbad, CA, USA; complete DMEM) at 37 °C in a humidified atmosphere containing 5% CO_2_. The establishment of RANKL-expressing HSC-2 HNSCC cells was described previously^18^. Among them, the RANKL-expressing R1 and R2 cells and the control C1 cells were used in this study.

### Antibodies and reagents

The antibodies to β-actin, IκBα (C-21), and NF-κB p52 (K-27) were obtained from Santa Cruz Biotechnology (Santa Cruz, CA, USA). The antibodies to NF-κB p65 and integrin α2 (VLA-2α) were from Abcam (Cambridge, MA, USA) and BD Biosciences Pharmingen (San Diego, CA, USA), respectively. The antibodies to phospho-p38 (pp38, T180/Y182), p38, phospho-Akt (pAkt, T308), pAkt (S473), Akt, phospho-JNK (pJNK, T183/Y185), JNK, phospho-ERK (pERK, T202/Y204), and ERK were purchased from Cell Signaling Technology (CST; Beverly, MA, USA). The antibodies to active integrin β1 (AIIB2) were obtained from the Developmental Studies Hybridoma Bank at the University of Iowa (Iowa City, IA, USA). The human recombinant osteoprotegerin (OPG, TNFRSF11B)/Fc chimera and TNF-α were from R&D Systems (Minneapolis, MN, USA) and PeproTech (Rocky Hill, NJ, USA), respectively. The NF-κB inhibitor BAY-11-7082 and p38 mitogen-activated protein kinase inhibitor SB203580 were obtained from Calbiochem (San Diego, CA, USA) and CST, respectively.

### Adhesion assay

The cell adhesion assay was performed essentially as previously described^64^. Briefly, a 96-well plate was coated with type I-C collagen (Col-I, Nitta Gelatin, Osaka, Japan), Matrigel (MG, BD-Discovery Labware, Bedford, MA, USA), laminin (LM, R&D Systems), and fibronectin (FN, Biomedical Technologies, Stoughton, MA, USA), or left uncoated. The cells (4 × 10^4^) were incubated for the indicated times at 37 °C in a humidified atmosphere containing 5% CO_2._ The bound cells were stained with 0.04% crystal violet, lysed with DMSO, and quantified by measuring the absorbance at 595 nm with a spectrophotometer (Bio-Rad microplate reader 550). Alternatively, the cells were prepared as described above, fixed in 3% paraformaldehyde for 15 min at room temperature, permeabilized with 0.1% Triton X-100 in PBS for 4 min at room temperature, and incubated with AlexaFluor 594-conjugated phalloidin (Invitrogen, 1:40 dilution) at 37 °C for 1 h. The cells were then imaged using a confocal laser-scanning microscope (FV-10i; Olympus, Tokyo, Japan); the cell size was measured using MetaMorph software (Molecular Devices, West Chester, PA, USA).

### Ethics

The tumor tissues from patients who had signed a written informed consent document were used for this study. This study was approved by the Institutional Review Board of Hokkaido University Hospital and was carried out in accordance with the Ethical Guidelines for Clinical Research.

### RNA isolation and RT-PCR

The total RNA isolation, first strand cDNA synthesis, and PCR were performed as previously described^65^. The sequences for the primers were as follows:

RANKL forward primer, 5′-TGGCACTCACTGCATTTATAGAATT-3′;

RANKL reverse primer, 5′-AGTTGAAGATACTCTGTAGCTAGGT-3′;

RANK forward primer, 5′-GGGAAAGCACTCACAGCTAATTT-3′;

RANK reverse primer, 5′-GCACTGGCTTAAACTGTCATTCTCC-3′;

integrin α2 forward primer, 5′-ACTTTGTTGCTGGTGCTCCT-3′;

integrin α2 reverse primer, 5′-CAAGAGCACGTCTGTAATGG-3′;

integrin α1 forward primer, 5′-GGTTCCTACTTTGGCAGTATT-3′;

integrin α1 reverse primer, 5′-AACCTTGTCTGATTGAGAGCA-3′;

integrin β1 forward primer, 5′-GAAGGGTTGCCCTCCAGA-3′;

integrin β1 reverse primer, 5′-GCTTGAGCTTCTCTGCTGTT-3′;

GAPDH forward primer, 5′-GAAATCCCATCACCATCTTCCAGG-3′; and

GAPDH reverse primer, 5′-CATGTGGGCCATGAGGTCCACCAC-3′.

Conventional RT-PCR was performed using a thermal cycler as follows: denaturation at 94 °C for 30 sec, annealing at 58, 57, 56, and 60 °C (for RANKL, RANK, integrin α2, and GAPDH, respectively) for 30 sec, and extension at 72 °C for 30 sec, followed by a final incubation at 72 °C for 10 min. The PCR products were subjected to 1% agarose gel electrophoresis, stained with ethidium bromide, and detected using an image analyzer (ATTO, Tokyo, Japan). Quantitative real-time PCR was performed as described^65^ using the same primer sets as for conventional RT-PCR.

### Immunoblotting

The cells were lysed in a solution containing 10 mM Tris-HCl (pH 7.4), 5 mM EDTA, 150 mM NaCl, 10% glycerol, 1% Triton X-100, 1% sodium deoxycholate, 0.1% SDS, 50 mM NaF, 1 mM Na_3_VO_4_, and complete (EDTA-free) protease inhibitors (Roche, Indianapolis, IN, USA) for 20 min on ice and clarified by centrifugation at 14,000 rpm for 10 min at 4 °C. The supernatants were subjected to SDS-PAGE, and the separated proteins were transferred to polyvinylidene difluoride membranes (Bio-Rad, Hercules, CA, USA). The membranes were incubated with the primary antibodies, followed by horseradish peroxidase-labeled secondary antibodies. The signals were developed using the ECL Western Blotting Detection Reagent (GE Healthcare, UK) and detected using an LAS-1000UV mini image analyzer (FUJIFILM, Tokyo, Japan).

### Transfection with siRNAs targeting the integrin α2 mRNA

The siRNAs targeting the integrin α2 mRNA (si Itga2) were obtained from QIAGEN (Valencia, CA, USA), and transiently transfected into the HSC-2-derived cells with the HiPerFect Transfection Reagent (QIAGEN) according to the manufacturer’s instructions. The AllStars Negative Control siRNA (si Ctrl, QIAGEN) was used as a control. After 72 h, integrin α2 protein levels were determined by immunoblotting, and the cells were subjected to various analyses.

### Dual-luciferase assay

C1, R1, and R2 cells (1.5 × 10^5^) were cultured in 12-well plates and co-transfected with 1 μg of pNF-kB-Luc (obtained from Dr. T. Taniguchi, the University of Tokyo, Japan) and 50 μg of pRL-TK (Promega, Madison, WI, USA) using Fugene HD (Roche). The luciferase assays were performed using the Dual-Luciferase reporter assay system (Promega) according to the manufacturer’s instructions. *Renilla* luciferase activity was used as an internal control.

### Chromatin immunoprecipitation (ChIP) assay

ChIP assay was performed using the Chromatin Immunoprecipitation (ChIP) Assay Kit (Upstate Biotechnology, Lake Placid, NY), according to the manufacture’s recommendation. C1 and R2 cells were fixed with 1% formaldehyde for 10 min at 37 °C, washed twice with ice-cold PBS containing 1 mM phenylmethylsulfonyl fluoride and Complete Protease Inhibitor Cocktail (Roche), and harvested by scraping and subsequent centrifugation. Cells were lysed in SDS Lysis Buffer for 10 min on ice, and chromatin was sheared by sonication (4 sets of 10-second pulses). After centrifugation, the supernatant were diluted (10-fold) in ChIP Dilution Buffer and pre-cleared with 50 μl of Salmon Sperm DNA/Protein A Agarose (50% slurry) for 30 min. After brief centrifugation, the supernatant was incubated in the presence or absence of an anti-NF-κB p65 antibody overnight at 4 °C with rotation. Immune complexes were collected with 60 μl of Salmon Sperm DNA/Protein A Agarose for one hour at 4 °C with rotation, and washed with the buffers listed in the order as follows: Low Salt Immune Complex Wash Buffer (once), High Salt Immune Complex Wash Buffer (once), LiCl Immune Complex Wash Buffer (once), and TE Buffer (twice). Immunoprecipitated histones were analyzed by immunoblotting using the anti-NF-κB p65 antibody. The immune complexes were also extracted in elution buffer (1% SDS and 0.1 M NaHCO_3_) and protein-DNA cross-links were reverted by heating at 65 °C for 4 h. DNA bound to the NF-κB p65 was analyzed by nested PCR using the following primers:

Itga2 promoter forward primer-1, 5′-CTGTGGTTTAGGGCTAGTGC-3′;

Itga2 promoter reverse primer-1, 5′-GTGGGCATATCCGACGGG-3′;

Itga2 promoter forward primer-2, 5′-GGGACTGGGGCATCTCCT-3′; and

Itga2 promoter reverse primer-2, 5′-GCATATCCGACGGGCGAG-3′.

### Live cell immunofluorescence and time-lapse microscopy

The adherent and suspension cells were incubated with an anti-active integrin β1 antibody (diluted 1:2000 with PBS containing 2% FBS) for 45 min on ice, washed with PBS, and further incubated with AlexaFluor 488-conjugated secondary antibody. For the suspension cells, the cells were re-plated on collagen-coated glass-bottom dishes. After fixation, the cells were observed under a fluorescence microscope. Of note, we performed this experiment after confirming that cell adhesion was not substantially affected in these conditions.

### Assessment of endocytosis

Endocytosis was evaluated as previously described^66^. Briefly, the cells were plated on collagen-coated glass-bottom dishes (35-mm diameter; Asahi Techno Glass, Tokyo, Japan), incubated with AlexaFluor-conjugated dextran (500 μg/ml, Invitrogen, for clathrin-independent endocytosis) or transferrin (500 μg/ml, Invitrogen, for clathrin-dependent endocytosis) for 10 min at 37 °C, washed extensively with PBS (Nissui, Tokyo, Japan), and then incubated in phenol red–free DMEM-F12 (Invitrogen). The visualized vesicles were extracted with the “granularity” function of MetaMorph software, and the fluorescence intensity was quantified.

### Three-dimensional (3D) culture and colony formation in collagen gels

The collagen gel cultures were essentially performed as previously described^67^, with some modifications based on the manufacturer’s protocol. Briefly, C1 or R2 cells (2 × 10^4^) were resuspended in 0.5 ml of DMEM containing 0.3% collagen (type I-A, Nitta Gelatin) with 10% FBS, and plated in 12-well dishes. After the collagen solution had gelled, 1 ml of complete DMEM was added to each well and changed every 7 days. After 21 days, the colonies were imaged and quantified.

### *In vivo* tumor formation in nude mice and TUNEL staining

Mouse husbandry and the animal experiments were approved by the Institutional Animal Care and Experiment Committee of Hokkaido University. The induction of tumor formation in the nude mice using the RANKL-expressing and control HNSCC cells, as well as the subsequent tissue manipulations, were described previously^18^. The obtained tumor tissue sections were subjected to TUNEL staining, observed under a microscope, and imaged.

### Statistical analyses

All data, unless otherwise specified, are expressed as the mean ± standard deviation (S.D.) and were subjected to one-way analysis of variance, followed by comparisons using Student’s *t-*test to evaluate the differences between the drug-treated and untreated samples. The *p* value in each test is represented by asterisks over the error bars in the figures and is described in the figure legends.

## Additional Information

**How to cite this article**: Yamada, T. *et al.* Receptor activator of NF-κB ligand induces cell adhesion and integrin α2 expression via NF-κB in head and neck cancers. *Sci. Rep.* **6**, 23545; doi: 10.1038/srep23545 (2016).

## Supplementary Material

Supplementary Movie S1

Supplementary Movie S2

Supplementary Information
