# Supplementary material for: Receptor activator of NF-κB ligand induces cell adhesion and integrin α2 expression via NF-κB in head and neck cancers
Source: Sci Rep. 2016 Mar 24;6:23545. doi: 10.1038/srep23545 (PMC4806381; doi:10.1038/srep23545)
Supplement: Supplementary Information [file srep23545-s3.pdf]

Supplementary Information

Receptor activator of NF-κB ligand induces cell adhesion and integrin α2 expression via NF-κB in head and neck cancers

Tamaki Yamada[1,2,3], Masumi Tsuda[4], Takanori Wagatsuma[1], Yoichiro Fujioka[1], Mari Fujioka[1], Aya O. Satoh[1], Kosui Horiuchi[1], Shinya Nishide[1], Asuka Nanbo[1], Yasunori Totsuka[3], Hisashi Haga[5], Shinya Tanaka[4], Masanobu Shindoh[2] & Yusuke Ohba[1]

Supplementary Movies S1, S2

Supplementary Movie S1. The trafficking of active integrin β1 in C1 cells, related to Fig. 6.

Supplementary Movie S2. The trafficking of active integrin β1 in R2 cells, related to Fig. 6.
